# Mapping Hypoxia in Renal Carcinoma with Oxygen-enhanced MRI: Comparison with Intrinsic Susceptibility MRI and Pathology

**DOI:** 10.1148/radiol.2018171531

**Published:** 2018-06-05

**Authors:** Ross A. Little, Yann Jamin, Jessica K. R. Boult, Josephine H. Naish, Yvonne Watson, Susan Cheung, Katherine F. Holliday, Huiqi Lu, Damien J. McHugh, Joely Irlam, Catharine M. L. West, Guy N. Betts, Garry Ashton, Andrew R. Reynolds, Satish Maddineni, Noel W. Clarke, Geoff J. M. Parker, John C. Waterton, Simon P. Robinson, James P. B. O’Connor

**Affiliations:** From the Centre for Imaging Sciences (R.A.L., J.H.N., Y.W., S.C., K.F.H., H.L., D.J.M., G.J.M.P., J.C.W.) and Division of Cancer Sciences (J.I., C.M.L.W., N.W.C., J.P.B.O.), University of Manchester, Manchester, England; Division of Radiotherapy and Imaging, The Institute of Cancer Research, London, England (Y.J., J.K.R.B., S.P.R.); Department of Pathology, Central Manchester University Hospitals NHS Foundation Trust, Manchester, England (G.N.B.); Department of Histology, CRUK Manchester Institute, Manchester, England (G.A.); Tumour Biology Team, The Breast Cancer Now Toby Robins Research Centre, The Institute of Cancer Research, London, England (A.R.R.); Department of Urology, Salford Royal Hospitals NHS Foundation Trust, Salford, England (S.M., N.W.C.); Bioxydyn Ltd, Manchester, England (G.J.M.P., J.C.W.); and Department of Radiology, The Christie NHS Foundation Trust, Manchester, England (J.P.B.O.).

## Abstract

**Purpose:**

To cross-validate T1-weighted oxygen-enhanced (OE) MRI measurements of tumor hypoxia with intrinsic susceptibility MRI measurements and to demonstrate the feasibility of translation of the technique for patients.

**Materials and Methods:**

Preclinical studies in nine 786–0-R renal cell carcinoma (RCC) xenografts and prospective clinical studies in eight patients with RCC were performed. Longitudinal relaxation rate changes (∆R1) after 100% oxygen inhalation were quantified, reflecting the paramagnetic effect on tissue protons because of the presence of molecular oxygen. Native transverse relaxation rate (R2*) and oxygen-induced R2* change (∆R2*) were measured, reflecting presence of deoxygenated hemoglobin molecules. Median and voxel-wise values of ∆R1 were compared with values of R2* and ∆R2*. Tumor regions with dynamic contrast agent–enhanced MRI perfusion, refractory to signal change at OE MRI (referred to as perfused Oxy-R), were distinguished from perfused oxygen-enhancing (perfused Oxy-E) and nonperfused regions. R2* and ∆R2* values in each tumor subregion were compared by using one-way analysis of variance.

**Results:**

Tumor-wise and voxel-wise ∆R1 and ∆R2* comparisons did not show correlative relationships. In xenografts, parcellation analysis revealed that perfused Oxy-R regions had faster native R2* (102.4 sec^–1^ vs 81.7 sec^–1^) and greater negative ∆R2* (−22.9 sec^–1^ vs −5.4 sec^–1^), compared with perfused Oxy-E and nonperfused subregions (all *P* < .001), respectively. Similar findings were present in human tumors (*P* < .001). Further, perfused Oxy-R helped identify tumor hypoxia, measured at pathologic analysis, in both xenografts (*P* = .002) and human tumors (*P* = .003).

**Conclusion:**

Intrinsic susceptibility biomarkers provide cross validation of the OE MRI biomarker perfused Oxy-R. Consistent relationship to pathologic analyses was found in xenografts and human tumors, demonstrating biomarker translation.

Published under a CC BY 4.0 license.

*Online supplemental material is available for this article.*

## Introduction

Hypoxia results from an imbalance between oxygen delivery and demand (1). Tumor hypoxia is an important negative prognostic factor in human cancers (2–4) and predicts treatment failure to both radiation therapy (5) and numerous chemotherapeutic agents (6). Interest in modifying or exploiting hypoxia has driven attempts to develop new treatments for use in combination with radiation therapy and chemotherapy (7). Effective development and delivery of these treatments requires imaging biomarkers that can rapidly identify and accurately assess the extent and spatial distribution of tumor hypoxia (8).

MRI techniques are being investigated for delivering translational biomarkers of hypoxia (9). Historically, interest has focused on intrinsic susceptibility imaging, which quantifies native values of effective transverse relaxation rate (R2*) and the change in R2* (∆R2*) induced by respiratory challenge with hyperoxic gas (10,11). However, there has been recent interest in quantifying the change in the longitudinal relaxation rate (R1) after inhalation of 100% oxygen (12). In this latter technique, referred to as oxygen-enhanced (OE) MRI, paramagnetic oxygen molecules dissolved in blood plasma, interstitial tissue fluid, or intracellular water can induce changes in R1 (∆R1) (13). Numerous studies (14–18) have reported an increase in R1 in well-oxygenated tissues after challenge with hyperoxic gas. In hypoxic tissue, the inhaled oxygen molecules bind preferentially to deoxygenated hemoglobin molecules, converting the paramagnetic deoxyhemoglobin to diamagnetic oxyhemoglobin. Hence, in hypoxic tumor subregions, there is no measurable positive ∆R1 (19).

**Figure 1:**
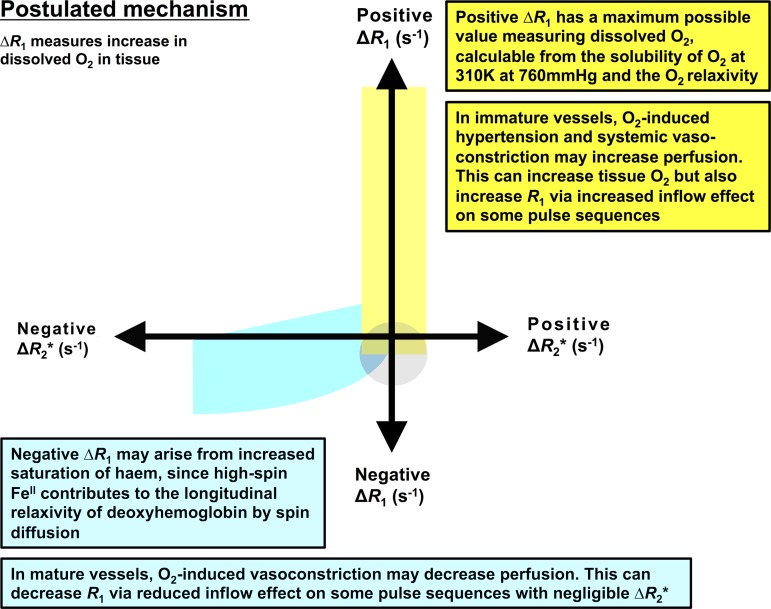
Schematic representation of postulated relationship between oxygen-induced change in R2* (∆*R*_2_*) and change in R1 (∆*R*_*1*_) MRI biomarkers of tumor hypoxia. It is postulated that change in R1 measures increase in dissolved oxygen in tissue. The theoretical relationships between voxels with different perfusion and oxygenation status are considered, along with physics confounds, such as inflow effects. Hypothetical distributions of voxels are indicated for normoxic (yellow), hypoxic (blue), and nonperfused (gray) tumor subregions.

We recently showed (20,21) in multiple preclinical xenograft models that tumor voxels with demonstrable perfusion but absent ∆R1 (oxygen refractory on R1 mapping referred to as perfused Oxy-R) represent a noninvasive signature of hypoxia at MRI. This finding suggests that OE MRI may have a translational benefit compared with R2*-based methods. Previous studies reported complex and nonlinear relationships between ∆R1 and ∆R2* in xenografts (22–26) or patient tumors (27) (Fig 1). However, to our knowledge, no cross-validation has been performed to evaluate whether perfused Oxy-R– and R2*-based biomarkers measure the same underlying tumor biology.

In our study, we hypothesized that tumor subregions identified as perfused Oxy-R would have faster native R2* and greater negative ∆R2*. We investigated this spatial relationship by using a renal cell carcinoma (RCC) xenograft model in which perfused Oxy-R was previously validated with pathologic measurement of hypoxia (21). We then performed a clinical study in patients with RCC tumors. The purpose was to cross-validate a T1-weighted OE MRI measurement of tumor hypoxia with T2*-weighted intrinsic susceptibility MRI measurements and to demonstrate the feasibility of translation of the technique into patients.

## Materials and Methods

AstraZeneca provided a salary to one author (K.F.H.). Two researchers provided consultancy to Bioxydyn (G.J.M.P. and J.C.W.). Authors who are not employees of or consultants for AstraZeneca and Bioxydyn had full control of inclusion of any data.

### Preclinical MR Data Acquisition

Experiments were performed in compliance with licenses issued under the UK Animals (Scientific Procedures) Act 1986, following local ethical review, and the United Kingdom National Cancer Research Institute guidelines for animal welfare in cancer research (28). The subcutaneous 786–0 RCC xenograft model (29), detailed MRI analysis, and pathologic analysis are described in Appendix E1 (online).

When the tumors were approximately 400 mm^3^, tumors were evaluated on a 7.0-T horizontal bore MRI system (Bruker, Ettlingen, Germany). Details of the anesthesia procedure are in Appendix E1 (online). After localization and shimming over the tumor, one axial imaging section was collected for all functional sequences. One R2* measurement was performed before and after the OE MRI sequence. Finally, dynamic contrast agent–enhanced (DCE) MRI (30) was performed while the mice inhaled 100% oxygen to define tumor perfusion. Functional imaging sequences (Table 1)were as follows:

1. Intrinsic susceptibility imaging: multiple gradient-echo images to derive R2*.
2. OE MRI: inversion recovery true-fast imaging with steady-state precession images to derive R1 at baseline and dynamically throughout the gas challenge. The dynamic series was performed for 10 minutes 40 seconds. This sequence is relatively insensitive to inflow effects.
3. DCE MRI: R1 was measured by using a modified true-fast imaging with steady-state precession sequence. After five baseline measurements, 0.1 mmol/kg bolus of gadopentetate dimeglumine (Magnevist; Bayer, Leverkusen, Germany) was injected intravenously at 2 mL/min by using a power injector. The dynamic series was acquired for 10 minutes 40 seconds.

**Table 1:**
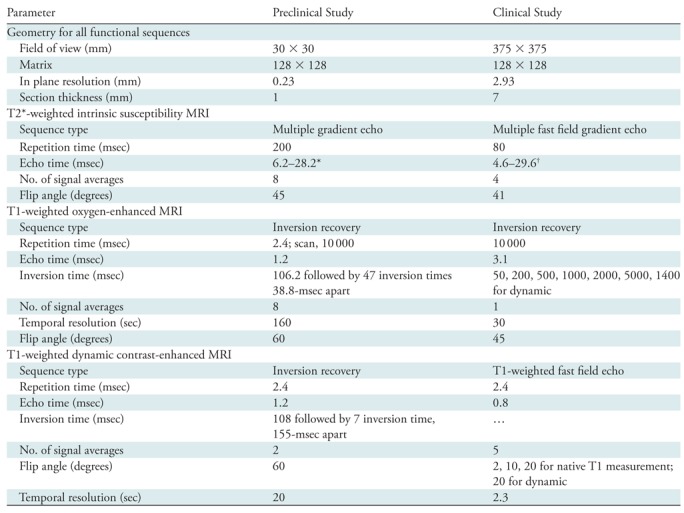
Imaging Sequence Details

*3.1-msec echo spacing

^†^5-msec echo spacing

### Clinical Study MRI Data Acquisition

Studies were performed after research ethics approval and institutional board review. Oxygen administration was not regarded as an investigational medicinal product study after consultation with the UK Medicines and Health Care Products Regulatory Agency. All patients gave fully informed written consent.

Patients with surgically resectable RCC (stage T1–T3, N0, M0) were recruited before nephrectomy. Subsequent immunohistochemical detection of the hypoxia-regulated gene glucose transporter 1 (*GLUT1*) provided an indirect assessment of tumor hypoxia (31).

Gas delivery (medical air or 100% oxygen) was at 15 L/min through a nonrebreathing mask (Adult EcoLite High Concentration Oxygen mask; InterSurgical, Berkshire, England). Oxygen concentration in the mask was monitored continuously (ML206 Gas Analyzer and Powerlab 8/35; ADInstruments, Oxford, England) and analyzed (LabChart version 7.3.4; ADInstruments).

Data were acquired by using a 1.5-T horizontal bore magnet (Philips Healthcare, Eindhoven, the Netherlands). Patients were imaged in the supine position with the scanner body resonator (Q body coil; Philips Healthcare) used in transmit and receive mode. After localization, one oblique coronal section was acquired that matched the coronal plane of the tumor-bearing kidney. Single R2* measurements were collected before and after the OE MRI sequence. Finally, DCE MRI was performed. Functional imaging sequences (summarized in Table 1) were as follows:

1. Intrinsic susceptibility imaging: multiple gradient-echo images to calculate R2*.
2. OE MRI: Inversion recovery half-Fourier rapid acquisition with relaxation enhancement images to calculate R1 at baseline and dynamically throughout the gas challenge. Dynamic images were used to quantify the temporal onset of R1 changes induced by switching between air and 100% oxygen (switch performed after nine baseline measurements).
3. DCE MRI: Native R1 was measured by using a variable flip angle spoiled gradient-echo sequence. Time varying R1 was determined by relating the time varying signal change to the native R1. After 14 baseline measurements, 0.1 mmol/kg bolus of gadoterate meglumine (Dotarem; Guerbet, Paris, France) was injected intravenously at 3 mL/min by using a power injector (Medrad Spectris MR; Bayer, Leverkusen, Germany), followed by a 20-mL saline flush.

### MRI Data Analysis

Regions of interest were drawn for tumors on the T2-weighted images by experienced operators (Y.J., with 13 years of preclinical experience, and Y.W., with 16 years of clinical experience) and transferred to the intrinsic susceptibility, OE MRI, and DCE MRI data for each tumor. Voxel-wise values of native R2*, oxygen-induced ∆R2*, and oxygen-induced ∆R1 were calculated for all parameters by using in-house software from which median values were derived.

For intrinsic susceptibility MRI, the voxel-wise native R2* was calculated by using the air-only data. Next, the ∆R2* was calculated as ∆R2* = R2* (O_2_) − R2* (air), where O_2_ is oxygen. At OE MRI, the voxel-wise ∆R1 was calculated as ∆R1 = R1 (O_2_) − R1 (air). At DCE MRI, voxels were classified as perfused or nonperfused (32).

**Figure 2:**
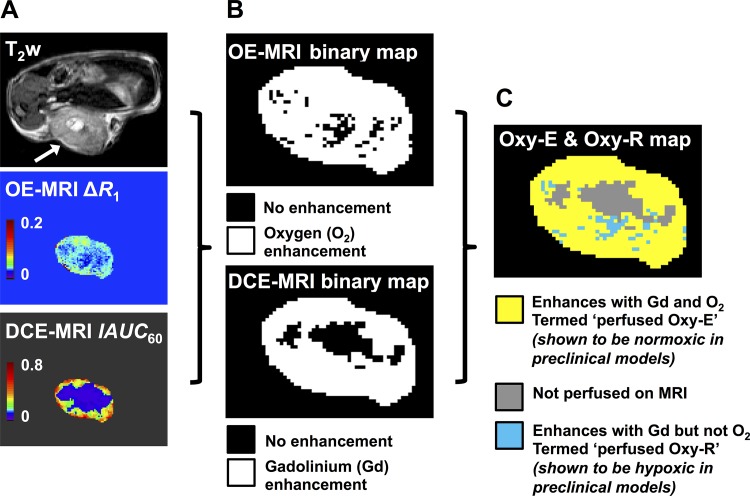
Schematic representation of analysis method used to define tumor subregions. *A*, For each preclinical and clinical tumor, a T2-weighted (T_2_w) anatomic image is used to define a region of interest (tumor; arrow), then oxygen-enhanced (OE) MR images and dynamic contrast-enhanced (DCE) MR images are analyzed to generate maps of change in longitudinal relaxation rate (∆R1) and the initial area under the gadolinium uptake curve from 0 to 60 seconds after injection of Gd-DTPA (IAUC_60_), respectively. *B*, The ∆R1 and IAUC_60_ data were binarized to produce binary enhancement maps, and then, *C*, combined to generate a map with three categories of voxels: perfused and OE, perfused but oxygen-refractory, and nonperfused.

For combined OE and DCE MRI analysis, voxels were classified as enhancing (hereafter, referred to as Oxy-E) or refractory (hereafter, referred to as Oxy-R) to oxygen challenge and then further subclassified as perfused or nonperfused by using DCE MRI data. From this, three subregions were defined: normoxic, composed of perfused Oxy-E voxels; hypoxic, composed of perfused Oxy-R voxels; and nonperfused voxels (Fig 2).

### Statistical Analysis

For both preclinical and clinical studies, median values of the MRI biomarkers (native R2*, oxygen-induced ∆R2*, and oxygen-induced ∆R1) and voxel-wise values were compared by using Spearman ρ. The relationship of the voxel-wise R2* and ∆R2* to perfused Oxy-R voxels, perfused Oxy-E voxels, and nonperfused voxels was evaluated by one-way analysis of variance.

The relationship of tissue pathology to MRI biomarkers (native R2*, oxygen-induced ∆R2*, oxygen-induced ∆R1, and perfused Oxy-R) in the 786–0-R xenografts was analyzed by using Spearman ρ. Clinical tumors were designated as either low or high in hypoxic fraction by semi-quantitative pathology, and the values of perfused Oxy-R were compared between these two groups by using the Student *t* test. In all cases, *P* values less than .05 were considered to indicate statistical significance following Bonferroni correction when multiple comparisons were tested.

## Results

### R1 Biomarkers But Not R2* Biomarkers Relate to Hypoxia in 786–0-R Xenographs

The relationships of MRI biomarkers of hypoxia and tissue pathologic assessment were determined in the 786–0-R xenografts. Median values of native R2* and oxygen-induced ∆R2* taken across the entire image did not correlate with the hypoxic fraction measured at pimonidazole adduct formation (Figs 3a, 3b). Hypoxic fraction was related to median values of oxygen-induced ∆R1 (ρ, −0.783; *P* = .013; Fig 3c) and the perfused Oxy-R fraction (ρ, 0.902; *P* = .002; Fig 3d).

**Figure 3a:**
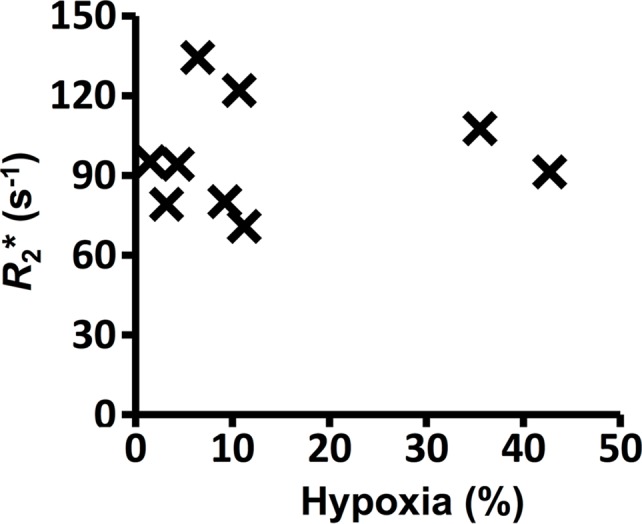
Graphs show the correlations between hypoxic fraction (expressed as a percentage and calculated from pimonidazole adduct formation immunohistochemistry images) and MRI biomarkers in 786–0-R tumors propagated in 8-week-old female C.B17-scid mice. Hypoxia did not correlate with **(a)** native R2* (*R*_2_*) or **(b)** oxygen-induced change in R2* (∆R_2_*), but it did correlate with **(c)** oxygen-induced change in R1 (∆R_1_) and **(d)** percentage of tumor perfused Oxy-R (nine mice for **a**–**c** and eight mice for **d**).

**Figure 3b:**
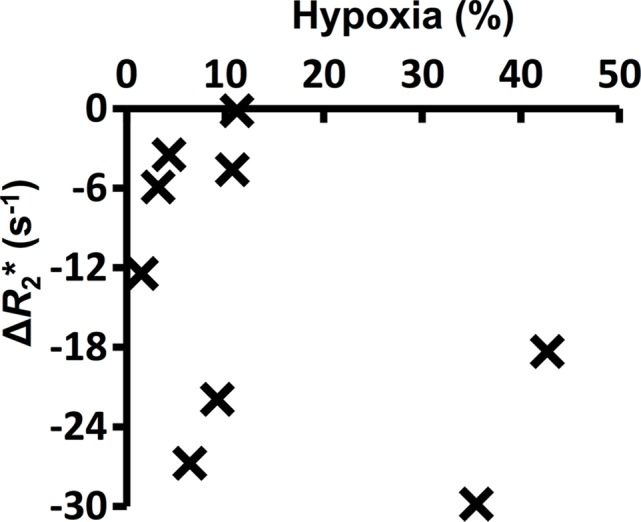
Graphs show the correlations between hypoxic fraction (expressed as a percentage and calculated from pimonidazole adduct formation immunohistochemistry images) and MRI biomarkers in 786–0-R tumors propagated in 8-week-old female C.B17-scid mice. Hypoxia did not correlate with **(a)** native R2* (*R*_2_*) or **(b)** oxygen-induced change in R2* (∆R_2_*), but it did correlate with **(c)** oxygen-induced change in R1 (∆R_1_) and **(d)** percentage of tumor perfused Oxy-R (nine mice for **a**–**c** and eight mice for **d**).

**Figure 3c:**
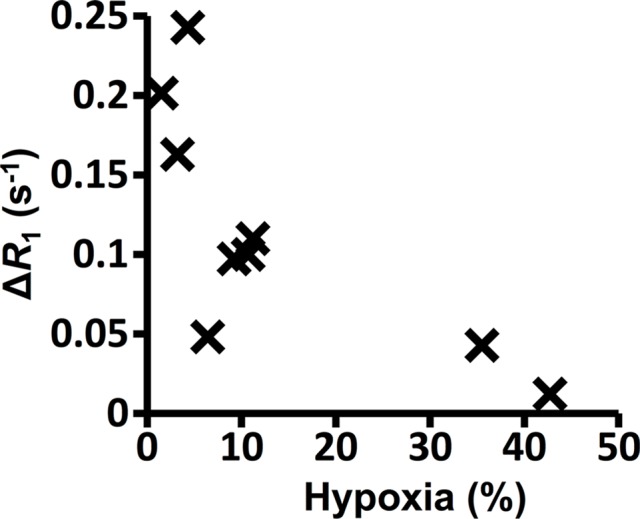
Graphs show the correlations between hypoxic fraction (expressed as a percentage and calculated from pimonidazole adduct formation immunohistochemistry images) and MRI biomarkers in 786–0-R tumors propagated in 8-week-old female C.B17-scid mice. Hypoxia did not correlate with **(a)** native R2* (*R*_2_*) or **(b)** oxygen-induced change in R2* (∆R_2_*), but it did correlate with **(c)** oxygen-induced change in R1 (∆R_1_) and **(d)** percentage of tumor perfused Oxy-R (nine mice for **a**–**c** and eight mice for **d**).

**Figure 3d:**
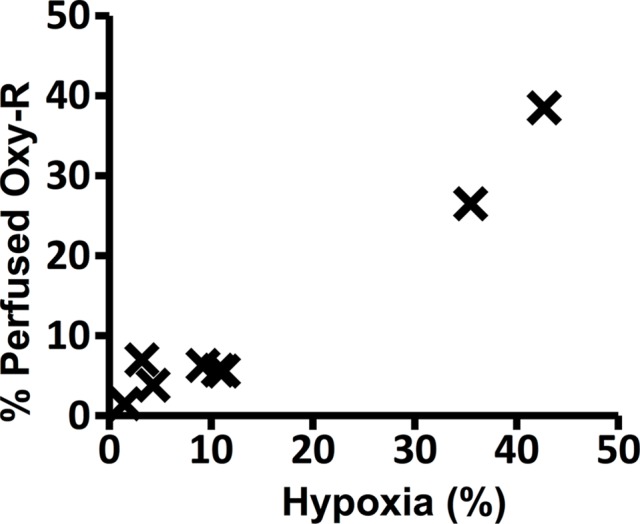
Graphs show the correlations between hypoxic fraction (expressed as a percentage and calculated from pimonidazole adduct formation immunohistochemistry images) and MRI biomarkers in 786–0-R tumors propagated in 8-week-old female C.B17-scid mice. Hypoxia did not correlate with **(a)** native R2* (*R*_2_*) or **(b)** oxygen-induced change in R2* (∆R_2_*), but it did correlate with **(c)** oxygen-induced change in R1 (∆R_1_) and **(d)** percentage of tumor perfused Oxy-R (nine mice for **a**–**c** and eight mice for **d**).

### Subregional Analysis Reveals the R2* and R1 Biomarker Relationship in 786–0-R Xenographs

The relationship between R2* and R1 biomarkers was compared. Initially, tumor-wise and voxel-wise analyses were investigated by following existing literature. Next, we used the combined OE and DCE MRI analysis to define the three subregions perfused Oxy-E tumor, perfused Oxy-R tumor, and nonperfused tumor.

***Tumor-wise analysis.—***Median values of native R2* and oxygen-induced ∆R2* were compared with median values of ∆R1 for each tumor. No significant correlations were observed.

***Voxel-wise analysis.—***Native R2* and ∆R1 did not have a significant relationship. In distinction, there was a highly significant but weak correlation between ∆R2* and ∆R1 (ρ, 0.230; *P* < .001; Fig E1 [online]). Voxels with greater negative gas-induced ∆R2* showed a smaller positive ∆R1, consistent with both being biomarkers of hypoxia. However, the relationship between ∆R2* and ∆R1 appeared complex and was not explained simply by the bimodal relationship predicted by the open L-shaped curve (24,27) (Fig 1).

***Parcellation analysis.—***We defined subregional analysis on the basis of the hypoxia biomarker perfused Oxy-R. This approach was chosen because we had previously validated Oxy-R as a hypoxia biomarker in this xenograft model (21). Three subregions were defined on the basis of combined OE MRI and DCE MRI signals (Fig 1). Native R2* and oxygen-induced ∆R2* were compared for each of these subregions. In all, 5815 voxels were included and analyzed, of which 488 (8.4%) were nonperfused; 4547 (78.2%) were defined as perfused Oxy-E, suggestive of a normoxic profile; and 780 (13.4%) were defined as perfused Oxy-R, suggestive of a hypoxic profile. Perfused Oxy-R voxels had faster native R2* (*P* < .001; Fig 4a) and greater negative hyperoxia-induced ∆R2* (*P* < .001; Fig 4b) than the perfused Oxy-E and nonperfused voxels. Example tumor parametric maps are shown with corresponding pathologic validation across the range of hypoxia measured (Fig 5).

**Figure 4a:**
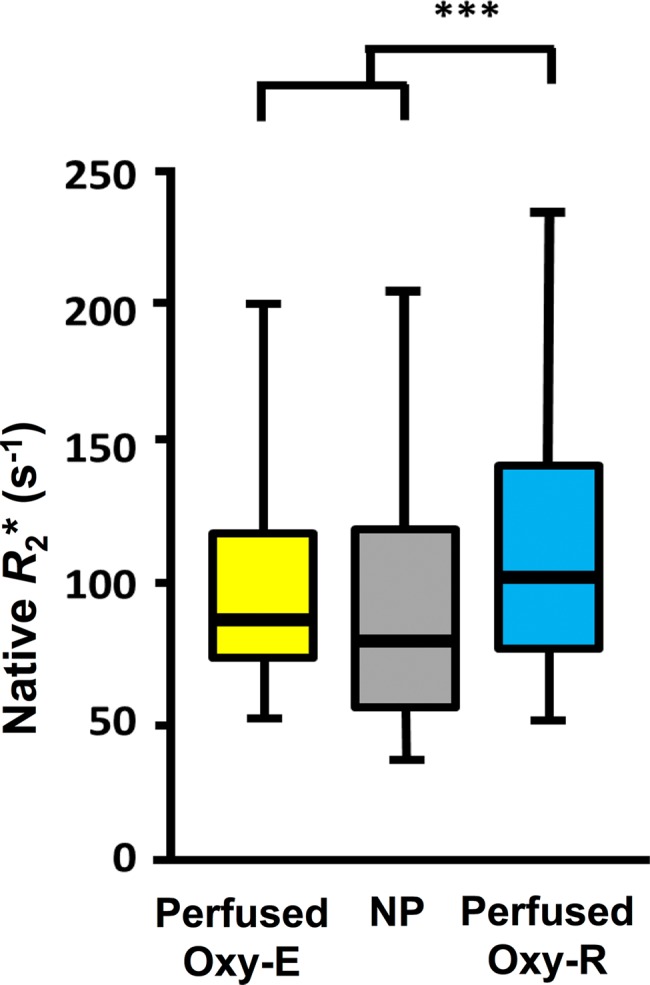
Box-and-whisker plots show relationship of voxel values of **(a)** native R2* (R_2_*) and **(b)** oxygen-induced change in R2* (∆R_2_*) to tumor subregions categorized by perfused Oxy-E, nonperfused (NP), and perfused Oxy-R in 786–0-R tumors propagated in 8-week-old female C.B17-scid mice (*n* = 8). Data are medians and interquartile range.

**Figure 4b:**
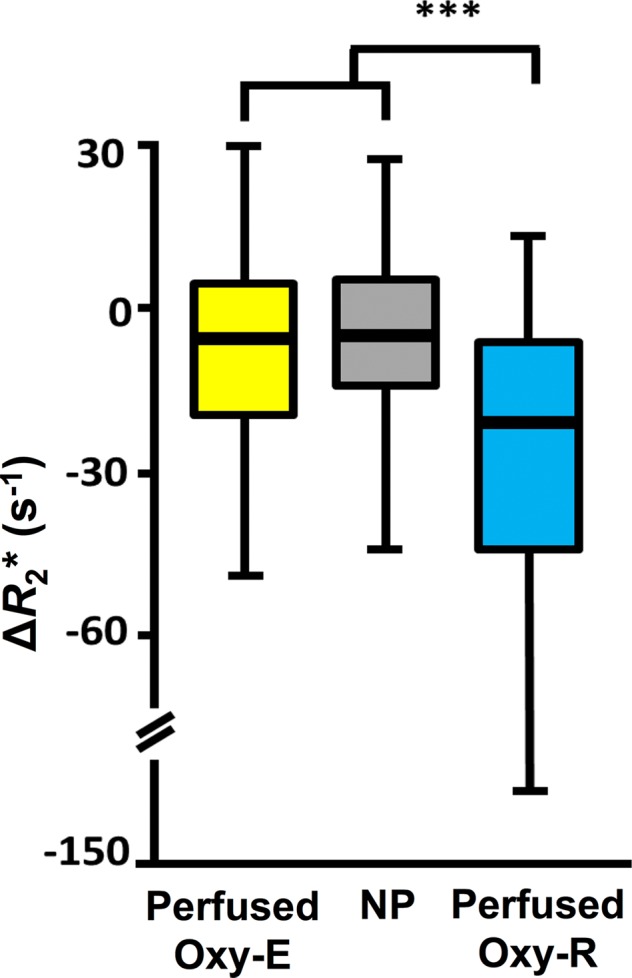
Box-and-whisker plots show relationship of voxel values of **(a)** native R2* (R_2_*) and **(b)** oxygen-induced change in R2* (∆R_2_*) to tumor subregions categorized by perfused Oxy-E, nonperfused (NP), and perfused Oxy-R in 786–0-R tumors propagated in 8-week-old female C.B17-scid mice (*n* = 8). Data are medians and interquartile range.

**Figure 5:**
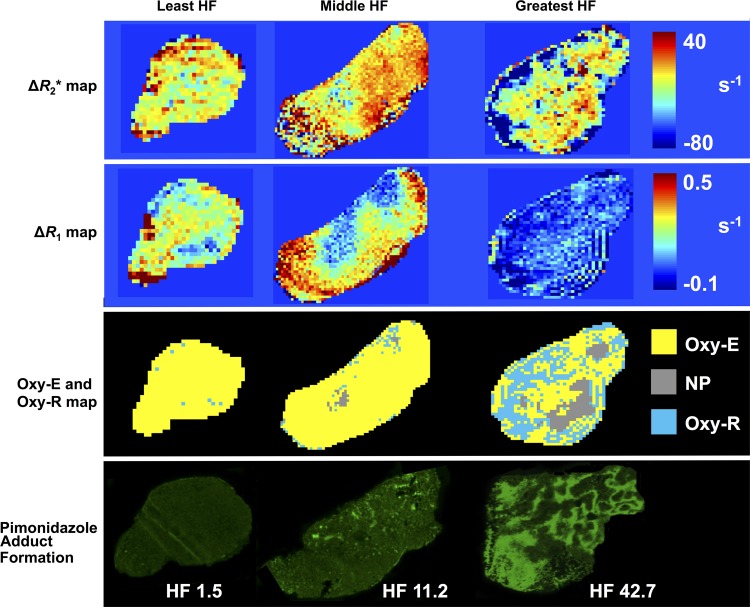
Representative parametric maps of change in R2* (*∆R*_2_*), change in R1 (*∆*R1), and combined oxygen-enhanced MRI and dynamic contrast-enhanced MRI (quantifying perfused Oxy-E, perfused Oxy-R, and nonperfused tumor) are shown for three 786–0-R tumors propagated in 8-week-old female C.B17-scid mice, showing least, middle and greatest hypoxic fractions (HF) measured by pimonidazole adduct formation.

### Technique Translation to Clinical Data

To test clinical translation, we recruited seven patients with clear cell RCC at radiologic assessment that was confirmed at subsequent histopathologic analysis (Table 2). The combined OE MRI and DCE MRI analysis requires reliable definition of voxels that are refractory to oxygen challenge. Data from the ML206 gas analyzer in all seven patients showed statistically significant increase in oxygen concentration to greater than 90% during gas challenge (sample trace in Fig E2 [online]).

**Table 2:**
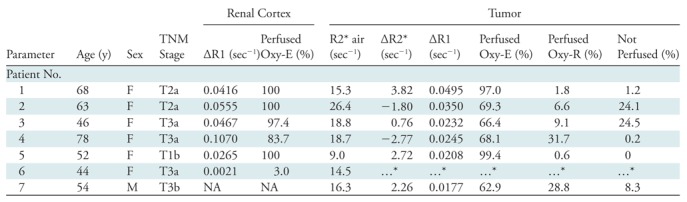
Patient Demographics, Stage, and Biomarker Values

Note.—∆R1 = change in R1, ∆R2* = change in R2*, F = female, M = male, NA = not available.

*Failed quality control for oxygen-enhanced MRI ∆R1 and ∆R2*.

As an additional quality control step, we evaluated the ∆R1 in the renal cortex to act as a positive control for oxygen delivery because positive ∆R1 has been consistently reported in multiple OE MRI studies (15–17) of the kidney. We evaluated renal cortex regions of interest for evidence of oxygen enhancement (Fig E3a [online]) and generated combined OE MRI and DCE MRI maps for these regions (Fig E3b [online]). These analyses showed that whereas all patients received high concentration oxygen, one patient failed to inhale the gas sufficiently to generate signal change in the renal cortex (only 3.0% of voxels were oxygen enhancing). All other patients with renal cortex in the field of view had significant positive ∆R1 in the renal cortex with between 83.7% and 100% (mean, 95.4%) of OE voxels (Table 2). Patient 7 had no normal kidney included in the field of view, but equivalent analysis of the spleen confirmed successful oxygen enhancement.

### Consistent Relationship between R2* and R1 Biomarkers Found in Human RCC Tumors

The analyses developed in the 786–0-R xenografts were applied to the patient data. Patient 6 tumor data were excluded because this patient failed quality control checks on the basis of renal cortex analysis. This tumor did not show significant oxygen enhancement in 84.3% of its voxels (Fig E4 [online]), which is consistent with a failure in gas delivery.

***Tumor-wise analysis.—***Median values of native R2* and gas-induced ∆R2* were compared with median values of ∆R1 for each tumor (*n* = 6). No significant correlations were observed.

***Voxel-wise analysis.—***Native R2* and ∆R1 did not have a significant relationship. However, there was a highly significant but weak correlation between ∆R2* and ∆R1 (ρ, 0.035; *P* < .001). Voxels with greater negative gas-induced ∆R2* showed a smaller change in R1 (Fig E5 [online]).

***Parcellation analysis.—***Native R2* and gas-induced ∆R2* were compared for each of three subregions, defined by their combined signals at OE MRI and DCE MRI. In total, 4112 voxels were measured, of which 436 (10.6%) were nonperfused, 2887 (70.2%) were defined as perfused Oxy-E suggestive of a normoxic profile, and 789 (19.2%) were defined as perfused Oxy-R suggestive of a hypoxic profile. Statistically significant differences were observed between the perfused Oxy-R voxels (predicted to be hypoxic) and both the perfused Oxy-E and the nonperfused voxels, with faster native R2* and a greater negative gas-induced ∆R2* in the perfused Oxy-R voxels (both *P* < .001) (Figs 6a, 6b).

**Figure 6a:**
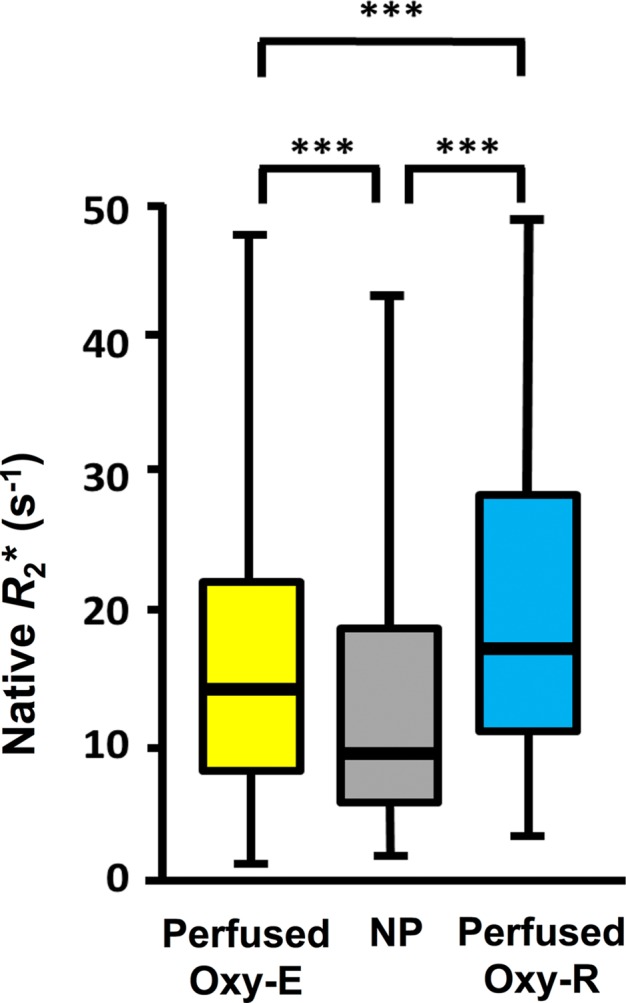
Box-and-whisker plots show relationship of voxel values of **(a)** native R2* (*R*_2_*) and **(b)** oxygen-induced change in R2* *(∆R*_2_*) to tumor subregions categorized by perfused Oxy-E, nonperfused Oxy-R, and perfused Oxy-R in patients with renal cell carcinoma (*n* = 6). Data are medians and interquartile range.

**Figure 6b:**
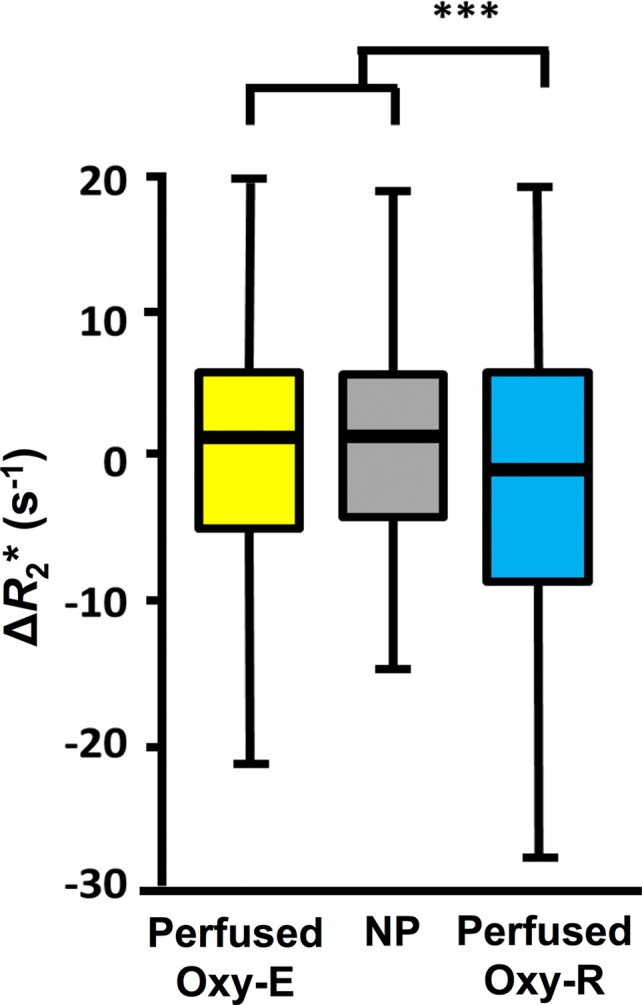
Box-and-whisker plots show relationship of voxel values of **(a)** native R2* (*R*_2_*) and **(b)** oxygen-induced change in R2* *(∆R*_2_*) to tumor subregions categorized by perfused Oxy-E, nonperfused Oxy-R, and perfused Oxy-R in patients with renal cell carcinoma (*n* = 6). Data are medians and interquartile range.

**Figure 7:**
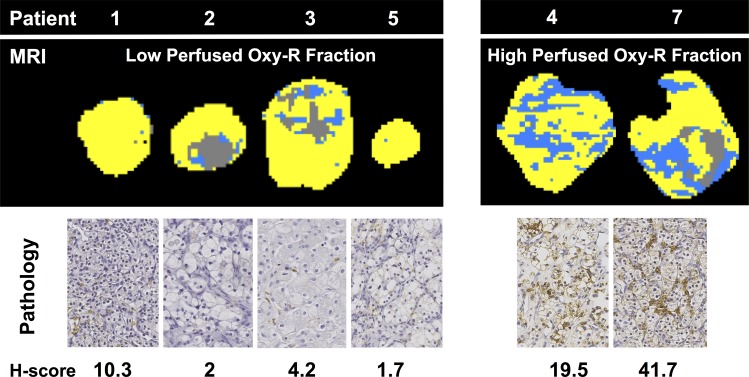
Relationship of perfused Oxy-R to hypoxia in patients with renal cell carcinoma. Parametric maps of perfused Oxy-E, perfused Oxy-R, and nonperfused subregions are shown for four patients with relatively low perfused Oxy-R fraction, with immunohistochemistry images for the hypoxia-regulated gene glucose transporter 1 used to obtain an indirect assessment of tumor hypoxia. For comparison, equivalent parameter maps and immunohistochemistry images (magnification, 40×) are shown for two patients with relatively high perfused Oxy-R fraction.

In an exploratory analysis, we scored tumor hypoxia by *GLUT1* staining. Although the study was not powered formally, the four tumors with MRI low hypoxic fraction (9.1%, 6.6%, 1.8%, and 0.6%) had *GLUT1* hypoxia scores of 4.2, 2, 10.3, and 1.7, respectively, whereas the two tumors with high MRI hypoxic fraction (31.7% and 28.8%) had *GLUT1* hypoxia scores of 19.5 and 41.7, respectively (Fig 7). Therefore, OE MRI helped to categorize the six patient tumors into two groups and helped to detect significant separation in *GLUT1* hypoxia score (*P* = .003).

## Discussion

There is a need to develop noninvasive biomarkers of tumor hypoxia. Potential applications include identifying patients who would benefit from modifying hypoxia before radiation therapy (7), identifying persistence of hypoxia during conventional treatment regimens (33), mapping targets for radiation boost or adaptive therapy (34), and monitoring changes in tumors treated with hypoxia-modifying therapies (7).

Whereas biomarkers have enormous potential in cancer, most failed to translate into practice-changing tools (35). Consequently, there is growing recognition that imaging biomarkers in development must undergo rigorous technical and biologic validation before they can be qualified as clinically useful (36).

Proton MRI methods are attractive because they are readily available and provide a cost-effective source of spatially localized information for various structural, functional, and molecular biomarkers of cancer (12). In this study, we sought to cross-validate oxygen-induced changes in R1, particularly the biomarker perfused Oxy-R, with the MRI biomarkers native R2* and oxygen-induced ∆R2*. This step is important because these different putative approaches to mapping hypoxia rely on different mechanisms and if measurements made by using these different approaches are mutually consistent, their validity is supported according to the Hill principle of coherence (37).

Initially, we compared four biomarkers of hypoxia measured tumor-wise (native R2*, oxygen-induced ∆R2*, oxygen-induced ∆R1, and perfused Oxy-R). The two R1-based biomarkers had significant relation to the pimonidazole-positive fraction in the well-vascularized but hypoxic 786–0-R RCC xenograft model, whereas native R2* and ∆R2* did not. At first, this supports the hypothesis that R1-based imaging biomarkers measure hypoxia, but it refutes the hypothesis that R2*- and R1-based imaging biomarkers identified the same hypoxic tumor subregions.

Next, we compared three biomarkers of tumor hypoxia with voxel-wise measurement (native R2*, oxygen-induced ∆R2*, oxygen-induced ∆R1). The relationship observed between oxygen-induced ∆R2* and oxygen-induced ∆R1 was weak and was not described fully by the open L-shaped model predicted by the literature (24,27). No relationship was observed between native R2* and oxygen-induced ∆R1.

Recognition that tumors are biologically heterogeneous suggests a need for an alternative investigative approach. We used OE MRI and DCE MRI to derive the biomarker perfused Oxy-R, which provides a hypoxic signature in tumor voxels (21). We then observed that the perfused Oxy-R tumor subregions had faster native R2* and greater negative ∆R2* after oxygen inhalation, as hypothesized, in both mouse xenograft tumors and in human RCCs.

This study emphasizes the substantial limitation of the use of summary value biomarkers to quantify tumor pathophysiologic features, such as hypoxia, that exhibit pronounced spatial variation (38). The data explain why R2*-based biomarkers may be insensitive indicators of hypoxia for some tumors. In this scenario, significant volumes of normoxic tissue and nonperfused tissue (collectively accounting for the vast majority of tumor tissue in most cancers) will mask the ability of native R2* and oxygen-induced ∆R2* to detect hypoxia. Indeed, over the last 2 decades, several studies (39–41) reported that hypoxic tumor tissue had faster native R2* and a greater negative ∆R2* after challenge with hyperoxic gas, whereas contrary findings (42) were reported elsewhere.

This study highlights the benefits of performing parallel preclinical and clinical experiments when evaluating translational potential of biomarkers, as recommended in the Cancer Research UK and European Organization for Research and Treatment of Cancer imaging biomarker roadmap (36). Preclinical studies allow rapid and early biologic validation, but have differences in data acquisition and analysis that could limit the ability of preclinical data to address clinical questions; (for example, the field dependence of R2* imaging results in a 22-fold higher effect at 7.0-T compared with 1.5-T). Despite this potential confound, the relationships between R2* and R1 biomarkers were equivalent in mice and humans: Voxels identified as having a hypoxic signature at combined OE MRI and DCE MRI (perfused Oxy-R voxels) had significantly faster native R2* and significantly greater oxygen-induced ∆R2* compared with voxels in tumor subregions with differing pathophysiology.

Further, the relative fraction of normoxic, hypoxic, and nonperfused tumor, defined by combined OE MRI and DCE MRI, was equivalent in mice and humans, indicating that the 786–0-R tumors were an appropriate model of heterogeneous hypoxia in clinical RCC tumors. Finally, exploratory analysis showed that two tumors with approximately 30% hypoxia measured by MRI had significantly higher *GLUT1* expression than the tumors with less than 10% hypoxia measured by MRI, providing evidence of equivalent imaging-pathology relationships in mice and human tumors.

Some study limitations should be recognized. First, although the findings of a xenograft study have been replicated in humans, the clinical sample size was small. Second, the one-way analysis of variance for voxel-level analysis does consider the clustering of voxels within the tumor. Third, voxel-wise data were pooled to perform the analyses on a cohort-level basis. Finally, whereas many results at the whole-tumor level were nonsignificant, this must be interpreted in light of the small sample sizes and consequent limited power.

In summary, these data use intrinsic susceptibility imaging and immunohistochemistry analysis to cross validate perfused Oxy-R as a regional biomarker of tumor hypoxia in mice and humans, providing strong rationale for further clinical translation of the biomarker. Further studies are required to test if the same relationships are observed between imaging and pathologic analysis and R1-based imaging and R2*-based imaging biomarkers in other tumor types, and to evaluate the value of perfused Oxy-R as a biomarker of prognosis, prediction of treatment response, and detection of response to therapy.

**Summary**

Intrinsic susceptibility imaging and immunohistochemistry analysis were used to validate a combined end point of oxygen enhancement plus MR-derived perfusion as a biomarker of tumor hypoxia in mouse and patient studies.

**Implications for Patient Care**

■ Oxygen-enhanced MRI is a feasible method to identify and map tumor hypoxia in patients.
■ Oxygen-enhanced MRI identifies spatial heterogeneity in tumor hypoxia, which may identify response to therapy and aid personalized radiation therapy treatment planning.

## APPENDIX

Appendix E1 (PDF)

## SUPPLEMENTAL FIGURES

Figure E1:

Figure E2:

Figure E3:

Figure E4:

Figure E5:
