# Supplementary material for: Mapping Hypoxia in Renal Carcinoma with Oxygen-enhanced MRI: Comparison with Intrinsic Susceptibility MRI and Pathology
Source: Radiology. 2018 Jun 5;288(3):739–47. doi: 10.1148/radiol.2018171531 (PMC6122194; doi:10.1148/radiol.2018171531)
Supplement: Appendix E1 (PDF) [file ry171531suppa1.pdf]

©RSNA, 2018
10.1148/radiol.2018171531

Appendix E1

Tumor Propagation

Cells established from a sunitinib refractory subcutaneous 786–0 RCC xenograft (786–0-R; derived from original parental line from ATCC, LCG Standards, Teddington, UK; purchased 2011) (1), were cultured in RPMI supplemented with 10% (v/v) fetal calf serum (Gibco, Life Technologies, Paisley, UK). Tumors were propagated by injecting 3×10^6 cells in 100 µl of sterile PBS into the flanks of 8-week-old female C.B17-scid mice under isoflurane anesthesia. Immediately prior to in vivo implantation, all cells tested negative for mycoplasma infection. Cell line authenticity was confirmed by PCR assessment of short tandem repeats profile.

Animals were housed in specific pathogen-free rooms in autoclaved, aseptic microisolator cages with a maximum of four animals per cage. Food and water were provided ad libitum. The mice were routinely monitored for appearance of palpable tumors. Animals were from a pilot study so no formal power calculation was performed. All animals that were entered into the protocol survived and gave analyzable data. Since all animals had one scan and no treatment, blinding and randomization was not required. No adverse effects were experienced by any animal.

Preclinical Anesthetic Method

Anesthesia was induced with a 10 mL/kg intraperitoneal injection of fentanyl citrate (0.315 mg/mL) plus fluanisone (10 mg/mL) (Hypnorm; Janssen Pharmaceutical Ltd, High Wycombe, UK), midazolam (5 mg/mL) (Hypnovel; Roche, Welwyn Garden City, UK) and sterile water (at 1:1:2 ratio) (2). Mice were positioned in a 3 cm birdcage coil on a custom built platform to isolate the tumor which was surrounded by dental paste (3M; Bracknell, UK) to minimize motion and susceptibility artifacts. Gas delivery (medical air or 100% oxygen) was continuous at 2 l/min through a nose piece. Warm air maintained animal core temperature at 37°C. Lateral tail vein cannulation was performed with a heparinized 27G butterfly catheter (Venisystems, Hospira, Royal Leamington Spa, UK) for intravenous administration of gadolinium contrast agent.

Preclinical Tumor Pathology Analysis

Hypoxic fraction was quantified in both 786–0-R xenografts and in patient tumors. For preclinical data, intraperitoneal injection of 60 mg/kg pimonidazole (Hypoxyprobe, Burlington, MA) was performed 55 minutes before 100% O_2 inhalation began. Tissue sections (5 µm) were obtained from snap frozen tumor material and scanned using fluorescent microscopy on a Panoramic 250 Flash system (3DHistech, Budapest, Hungary). Pimonidazole binding was determined using Hypoxyprobe-1 (Hypoxyprobe, Burlington, MA), a mouse-monoclonal, followed by rabbit antimouse-fluorescein isothiocyanate (FITC) conjugated secondary antibody (excitation 488 nm/emission 525 nm).

Clinical Tumor Pathology Analysis

Whole nephrectomy was performed and specimens were transferred to the pathology laboratory within 30 minutes. Tissue sections (4 µm) were obtained from formalin fixed paraffin embedded tumor material. Immunohistochemistry for the hypoxia-regulated gene glucose transporter 1 (GLUT1) staining was performed using a rabbit polyclonal antihuman GLUT1 (GT-12A; Alpha Diagnostics International, San Antonio, USA) at 10 µg/mL concentration, detected using rabbit Envision Plus HRP (Dako K4003) and visualized (brown stain) using DAB. A hypoxia score (H score) was generated by an experienced consultant clinical pathologist (GNB). All pathology images were scanned using a *Leica SCN400* slide scanner microscope (Leica Microsystems, Milton Keynes, UK) at 40× magnification.

Image Analysis

Comparable analyses were performed for both preclinical and clinical data. R_2* values were calculated using an exponential fit to the data, for both preclinical and clinical tumors.

In preclinical data analysis, the R_1(air) was calculated as the average of the first two R_1 maps. The $R_1(O_2)$ was derived from the R_1 map acquired during oxygen breathing. Voxels were defined as oxygen enhancing if the ΔR_1 > than (2 × voxel native R_1 x cohort coefficient of variation (CoV) for the R_1; where the CoV was determined from two air-breathing R_1 acquisitions. In clinical data analysis used a least squares fit to the multiple inversion time data to define voxel R_1. The R_1(air) was derived from the average of the first nine R_1 maps. The $R_1(O_2)$ was derived from the average of the last nine time points at the end of the oxygen breathing phase. Voxels were defined as oxygen enhancing by performing a *t* test on the R_1 values from the air and oxygen phases.

Preclinical analysis derived the model free parameter initial area under the curve was calculated from 0 to 60 seconds ($IAUC_{60}$; units mmol.kg^{-1}) (3) and voxels with $IAUC_{60} > 0$ were considered enhancing. Clinical analysis compared signal intensity before (first 14 time points) and after (time points 100–155) contrast agent injection, with significant enhancement for each voxel determined by a *t* test.

References

1. Bridgeman VL, Wan E, Foo S, et al. Preclinical Evidence That Trametinib Enhances the Response to Antiangiogenic Tyrosine Kinase Inhibitors in Renal Cell Carcinoma. Mol Cancer Ther 2016;15(1):172–183 .

2. Burrell JS, Walker-Samuel S, Baker LC, et al. Exploring ΔR(2) * and ΔR(1) as imaging biomarkers of tumor oxygenation. J Magn Reson Imaging 2013;38(2):429–434 .

3. Evelhoch JL. Key factors in the acquisition of contrast kinetic data for oncology. J Magn Reson Imaging 1999;10(3):254–259 .

Figure E1: Scatterplot of voxel-wise ΔR_2* and ΔR_1 in 786–0-R tumors. Data are summated for all tumors (n = 9).

Figure E2: Quality assurance through measuring mask gas concentration. ML206 Gas Analyzer was used to sample the percentage O_2 and CO_2 detected in the nonrebreathing mask. Typical data from one patient is shown during (a) air breathing and (b) 100% oxygen breathing, using analysis in LabChart version 7.3.4.

Figure E3: Quality assurance of clinical OE-MR imaging data. (a) Parametric maps from each patient renal cortex region of interest shows that the majority of voxels are perfused Oxy-E in patients 1–5. Nearly all voxels are perfused Oxy-R in patient 6, indicating failure of oxygen challenge. (b) Median ΔR_1 for each patient region of interest were calculated and an average curve for patients 1–5 show positive ΔR_1 after switch from air to 100% oxygen (error bars are standard erro of the mean). The curve for patient 6 shows no positive enhancement.

Figure E4: MR imaging data for a patient who failed quality assurance. Quality assurance identified lack of enhancement in a control organ (renal cortex). The tumor combined OE-MR imaging and DCE-MR imaging parameter map shows negligible oxygen-enhancement (perfused Oxy-E), as expected. This patient data were not included in the subsequent analyses.

Figure E5: Scatterplot of voxel-wise ΔR_2^* and ΔR_1 in patients with RCC. Data are summated for all tumors (n = 6).
