# Supplementary figures and images for: Mapping Hypoxia in Renal Carcinoma with Oxygen-enhanced MRI: Comparison with Intrinsic Susceptibility MRI and Pathology
Source: Radiology. 2018 Jun 5;288(3):739–47. doi: 10.1148/radiol.2018171531 (PMC6122194; doi:10.1148/radiol.2018171531)

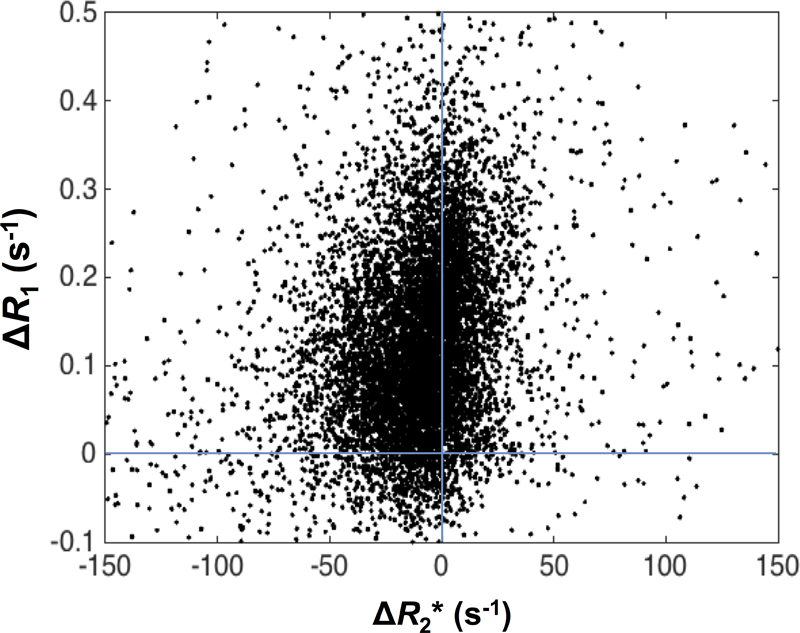

Supplement: Figure E1: [file ry171531suppf1.jpg]

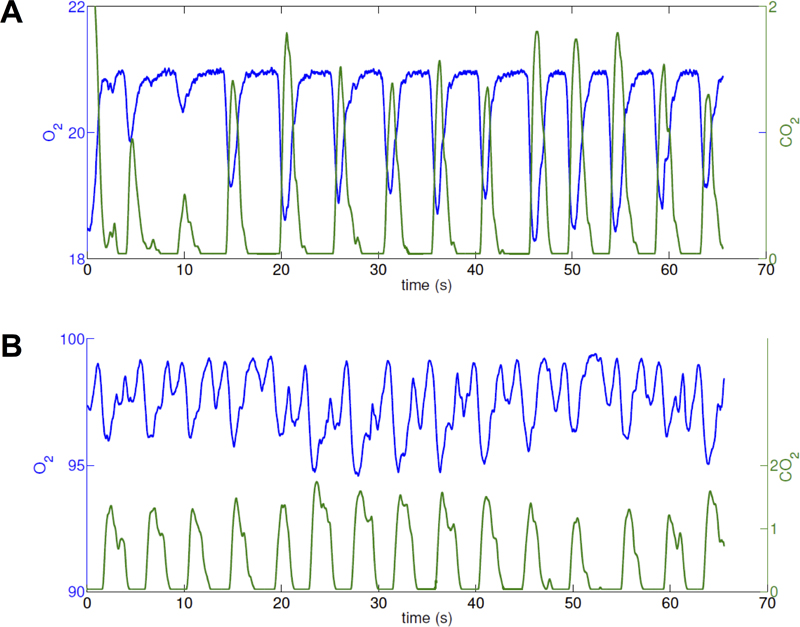

Supplement: Figure E2: [file ry171531suppf2.jpg]

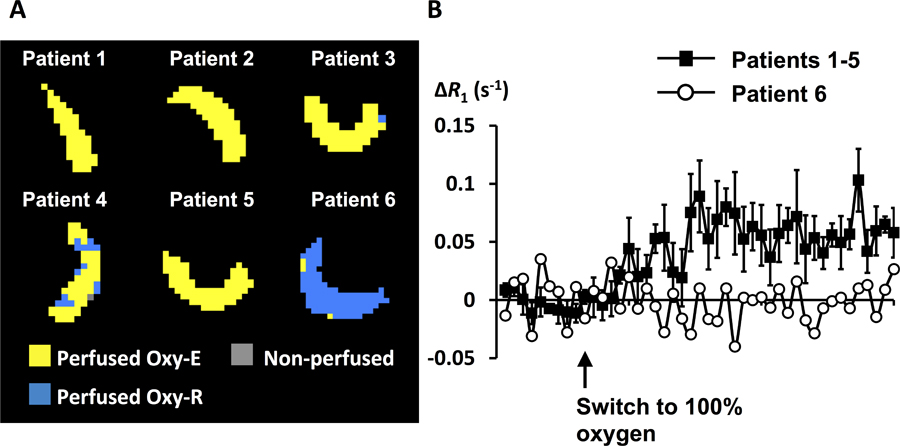

Supplement: Figure E3: [file ry171531suppf3.jpg]

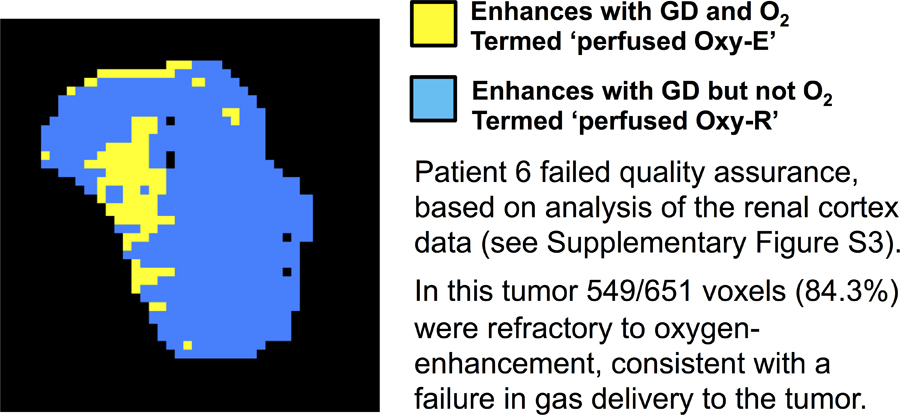

Supplement: Figure E4: [file ry171531suppf4.jpg]

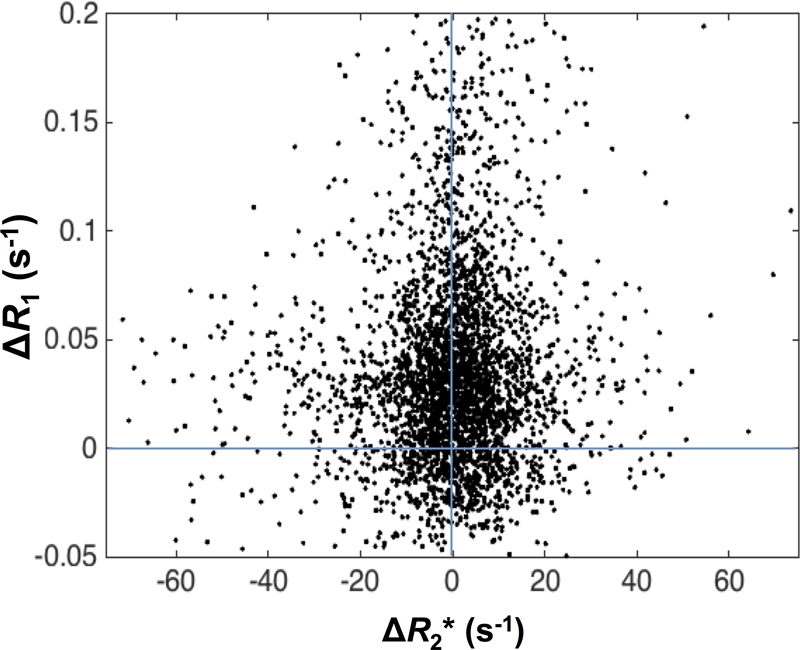

Supplement: Figure E5: [file ry171531suppf5.jpg]
